# Carbonate Chemistry and Isotope Characteristics of Groundwater of Ljubljansko Polje and Ljubljansko Barje Aquifers in Slovenia

**DOI:** 10.1155/2013/948394

**Published:** 2013-12-21

**Authors:** Sonja Cerar, Janko Urbanc

**Affiliations:** Geological Survey of Slovenia, Department of Hydrogeology, Dimičeva ulica 14, SI-1000 Ljubljana, Slovenia

## Abstract

Ljubljansko polje and Ljubljansko Barje aquifers are the main groundwater resources for the needs of Ljubljana, the capital of Slovenia. Carbonate chemistry and isotope analysis of the groundwater were performed to acquire new hydrogeological data, which should serve as a base for improvement of hydrogeological conceptual models of both aquifers. A total of 138 groundwater samples were collected at 69 sampling locations from both aquifers. Major carbonate ions and the stable isotope of oxygen were used to identify differences in the recharging areas of aquifers. Four groups of groundwater were identified: (1) Ljubljansko polje aquifer, with higher Ca^2+^values, as limestone predominates in its recharge area, (2) northern part of Ljubljansko Barje aquifer, with prevailing dolomite in its recharge area, (3) central part of Ljubljansko Barje aquifer, which lies below surface cover of impermeable clay and is poor in carbonate, and (4) Brest and Iški vršaj aquifer in the southern part of Ljubljansko Barje with higher Mg^2+^ in groundwater and dolomite prevailing in its recharge area. The radioactive isotope tritium was also used to estimate the age of groundwater. Sampled groundwater is recent with tritium activity between 4 and 8 TU and residence time of up to 10 years.

## 1. Introduction

The Ljubljansko polje and Ljubljansko Barje aquifers are the two most important groundwater sources for Ljubljana, the capital city of Slovenia, and its surrounding area. Groundwater is exploited in five pumping stations: Šentvid, Kleče, Hrastje, and Jarški prod from Ljubljansko polje aquifer, and Brest from Ljubljansko Barje aquifer. The natural conditions of the study area are characterized by high vulnerability of the aquifers, high velocities of groundwater flow and pollutant transport, and strong interconnection between the surface water and groundwater [1].

Groundwater chemistry is an important factor determining its use for domestic, irrigation, and industrial purposes. It is being controlled greatly by interaction with mineral composition of the aquifer through which it flows. In any area, groundwater has its unique chemistry due to several processes like soil/rock-water interaction during recharge and groundwater flow, prolonged storage in the aquifer, dissolution of mineral species, and so forth [2]. Reactions between groundwater and rock minerals have a significant influence on water quality and are also useful for understanding the genesis of groundwater [3]. The groundwater chemistry of alluvial aquifers typically reflects the chemistry of the dominant rock type in the surrounding recharge areas. Calcium, magnesium, and bicarbonate are the dominant ions present in groundwater in the research area. Carbonate-rich rocks such as limestone, dolomitic limestone, and dolomite are the major starting materials for carbonate weathering. The available carbonates in these rocks are dissolved and added to the groundwater system by way of rainfall and irrigation infiltration and groundwater movement [4, 5].

Groundwater quality of the aquifers is also influenced by anthropogenic factors such as industry, agriculture, population activities, traffic, waste disposal, and unregulated sewage system [6]. Thus, the geochemistry of a groundwater reflects a sometimes complex history of its flow path through various rock types in combination with anthropogenic influences [7].

The use of major ions as natural tracers [8] has become a very common method to delineate flow paths in aquifers [9]. The main chemical parameters that describe the groundwater carbonate equilibrium are calcium (Ca^2+^), magnesium (Mg^2+^), their molar ratio (Ca/Mg), and hydrogen carbonate (HCO_3_^−^). Usually the molar ratio between calcium and magnesium in groundwater depends on lithological composition of groundwater recharge areas. That is, if the molar ratio Ca/Mg is equal to 1, dissolution of dolomite prevails, while higher ratio indicates a greater contribution of calcite minerals dissolution [10].

Also the stable isotopes of oxygen and hydrogen are very good indicators for determining recharge areas of aquifers, since the values of isotopes remain constant in low temperature aquifers [11]. Altitude and continental effects have the main influence on stable isotopes in groundwater. At higher altitudes, where the average temperatures are lower, precipitations are isotopically depleted [12], with depletion rate of oxygen-18 around 0.3‰  per 100 m. Therefore, the altitude effect is very useful for distinguishing groundwater recharge areas at higher and lower altitudes [11]. The effect  is the observation that meteoric water is more depleted of oxygen-18 farther from the source (ocean) of the water vapor and is called continental isotope effect [13]. However, in smaller hydrologic basins the continental effect is negligible, while in larger systems this effect must be taken into consideration. Tritium is the radioactive isotope of hydrogen, with a half-life of 12.4 years, that enters the hydrological cycle via precipitation. Its presence in groundwater provides evidence for an active recharge. As it is part of the water molecule, it offers the only direct water dating method available [11]. Given that groundwater tritium content values vary both spatially and temporally, it is important to establish the closest precipitation measurement point to provide a reference to estimating groundwater recharge and travel times [14].

Chemical and isotopic composition of groundwater of the aquifers have been a case of study since 1997 [15–18]. Previous studies have focused only on researching individual aquifers. According to Jamnik et al. [19] groundwater of Ljubljansko polje aquifer is partly recharged with groundwater of Ljubljansko Barje aquifer, so the focus of this research was to study interaction between those two aquifers. The goal of the presented research, however, was to acquire new hydrogeological data and knowledge about both aquifers, which would serve as a base for improvement of hydrogeological conceptual models of both aquifers. In this scope we performed an extensive hydrochemical and isotope analysis of the groundwater, including parameters relevant for understanding mechanisms of aquifer recharge, groundwater retention time in the aquifer, and finally discharge from the aquifer to the Sava River water body.

### 1.1. Characteristics of the Study Area

**Figure 1:**
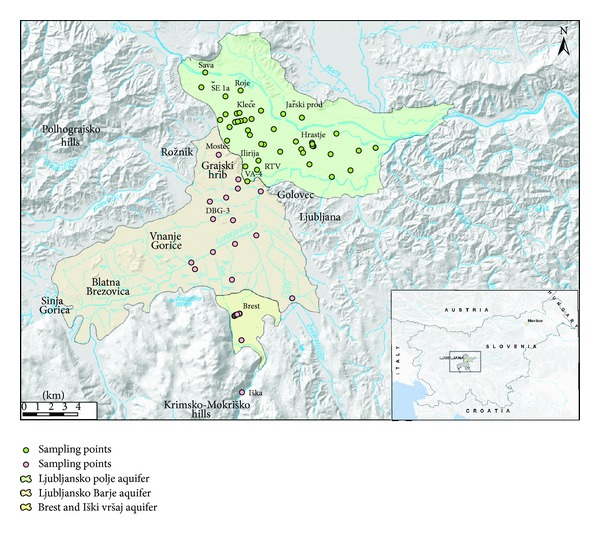
Location map of study area and groundwater sampling points.

The study area, situated in the central part of Slovenia, consists of two aquifers: Ljubljansko polje and Ljubljansko Barje. Ljubljansko polje lies on the northern part of Ljubljana, and Ljubljansko Barje lies on the southern part, and they are separated by the hills of Golovec, Grajski hrib, and Rožnik (Figure 1).

From a geological point of view the areas of Ljubljansko polje and Ljubljansko Barje are depressions formed by tectonic subsidence and gradual filling by alluvial and lacustrine sediments [20, 21]. The hills of Golovec, Grajski hrib, and Rožnik represent outcrops of the Carboniferous and Permian basement of the depression which was uplifted above the surface of the younger sediments. Sinja Gorica, Blatna Brezovica, Vnanje Gorice, and other hills in the southern part of Ljubljansko Barje are of similar origin. In the southern and western part of Ljubljansko Barje the basement consists of permeable Mesozoic limestone and dolomite, while elsewhere the basement consists of above mentioned Carboniferous and Permian mudstone, quartz sandstone, and conglomerate characterized by very low hydraulic conductivity.

Filling of depression with sediments was very intense in Pleistocene, when Sava River transported material from alpine glaciers to Ljubljansko polje [21]. The sediments are composed of well permeable gravel and sand beds with lenses of conglomerate. Due to considerable thickness, which exceeds 100 m in the deepest parts, and good permeability, this sandy-gravelly aquifer contains significant quantities of groundwater. The unconfined Ljubljansko polje aquifer is recharged by Sava River and local precipitations percolating through the vadose zone. In the northwestern part of Ljubljansko polje, area of Roje and Tomačevo, the aquifer is recharged by Sava River. Groundwater is drained back to the Sava River in the southeastern parts of Ljubljansko polje.

Ljubljansko Barje consists of alternating alluvial and lacustrine sediments. Alluvial sediments were brought to Ljubljansko Barje by rivers and creeks from southerly lying Krimsko-Mokriško hills. Gravel of Iška, Borovniščica, and Želimeljščica Rivers extends far to the north, where it reaches gravel of Gradaščica and Glinščica Rivers, and also Sava that flowed on the southern side of Rožnik hill till the end of last glacial. Pleistocene and Holocene alluvial sediments include interbedded lacustrine sediments. Lenses of clay, sand, and peat are common. Gravel is often silty or clayey. Surface in central parts of Ljubljansko Barje is covered by clayey silt with numerous remains of gastropods. Overall thickness of sediments is up to 170 m [20].

The near-surface Holocene aquifers of Ljubljansko Barje are recharged directly from the rainfall and surface streams, while deeper sand and gravel aquifers, formed on permeable limestone and dolomite basement, are fed by water from the extensive karst catchment south and west of Ljubljansko Barje. Due to the heterogeneity of sediments, hydrogeological conditions on Ljubljansko Barje are more complicated than on Ljubljansko polje. Aquifers of Ljubljansko Barje are situated in dolomite basement and in gravel beds. The last are separated by less permeable layers and therefore contain artesian groundwater. Groundwater of Ljubljansko Barje discharges to Ljubljanica River and to Ljubljansko polje through the narrow passage between Grajski hrib and Rožnik and Dravlje valley [19, 20].

## 2. Materials and Methods

### 2.1. Groundwater Sampling and Analysis

Groundwater samples were collected twice (autumn 2010 and spring 2011) on each sampling location during the groundwater base flow condition. In total, 44 samples were collected from Ljubljansko polje aquifer and 25 from Ljubljansko Barje aquifer. The sample sites in both aquifers comprise 28 water-supply pumping stations, 5 industry wells, 2 private wells, 29 boreholes, and 5 surface waters. The isotopic composition of oxygen (*δ* ^18^O) and tritium activity (^3^H) have been monitored in monthly precipitation at Geological Survey in Ljubljana since 2010.

Standard procedures for sampling, transport, and storage of groundwater samples were in accordance with the ISO standards [22–24]. Water samples from wells and boreholes were taken using a Grundfos MP-1 pump at a pumping rate of 0.2 L/s. During pumping the field parameters (electrical conductivity, pH, and water temperature) were measured by WTW pH/Conductivity measuring instrument pH/Cond 340i SET. The groundwater samples were collected when the measured field parameters of fresh groundwater at the object were stable.

Groundwater samples were taken in polyethylene bottles. For relevant major ions (Ca^2+^, Mg^2+^, and HCO_3_^−^) and radioactive isotope tritium (^3^H) 1 L of water sample was collected, and for stable isotopes of oxygen (^18^O/^16^O) 0.1 L of water sample was collected.

Major ions were analyzed in laboratory of public utility Vodovod-Kanalizacija d.o.o. Ljubljana. Ion chromatography method (IC Metrohm) was used for determination of different major ions which are expressed in mg/L. The precision of measurements is expressed as relative standard deviation (RSD) which is 0.46% for Ca^2+^ and 1.03% for Mg^2+^. The values of limit of detection (LOD) are 4.0 mg/L for Ca^2+^ and 2.0 mg/L for Mg^2+^, respectively. Analytical uncertainty for Ca^2+^ determination was 2∗(0.08∗4.0 + 0.02∗*C*_sample_) mg/L and for Mg^2+^  2∗(0.08∗2.0 + 0.02∗*C*_sample_) mg/L, where *C*_sample_ means concentration of the sample [25].

Groundwater samples for radioactive isotope tritium (^3^H) were analyzed at Jožef Stefan Institute in Ljubljana by the electrolytic enrichment method. Tritium concentrations are expressed as tritium units (TU), where 1 TU defines the presence of one tritium atom in 10^18^ atoms of hydrogen (H) [14]. Limit of detection is reported according to Curie's criteria [26] and it is determined for each water sample individually, with values ranging between 0.41 and 1.18 TU. The reported uncertainties are calculated in accordance with GUM (1995) and are expressed as value of the combined standard uncertainty of the specific activity and correspond to the confidence interval with a 68% confidence [27].

Groundwater samples for stable isotopes of oxygen (^18^O/^16^O) were analyzed at the laboratory of hydroisotop GmbH in Germany. The mass spectrometer Finnigan MAT 250 was used to determine the isotope ratio of stable isotopes. The stable isotope ratio is expressed in delta notation (*δ*) in parts per thousand (‰), which compares the ratio between heavy and light isotopes of a sample to that of a reference standard Vienna Standard Mean Ocean Water (V-SMOW) [11]. The maximum reported analytical uncertainty for *δ* ^18^O is ±0.15‰  and the accuracy of the instrument is better than 0.03‰.

## 3. Results and Discussion

### 3.1. Chemical and Isotopic Characteristics of Groundwater of Ljubljansko Polje and Ljubljansko Barje Aquifers

On the basis of the chemical and isotope analyses we acquired good insight into the hydrochemical processes (rock-water interaction) occurring in both aquifers of the research area. New data on natural chemical composition of groundwater and its age were obtained.

**Figure 2:**
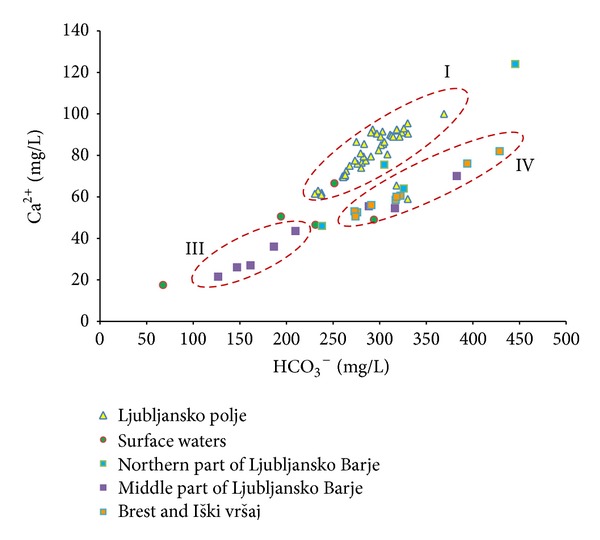
Relationship between HCO_3_^−^ and Ca^2+^ in groundwater of Ljubljansko polje and Ljubljansko Barje aquifers. Roman numerals represent groups of groundwater from (I) Ljubljansko polje aquifer, (III) middle part of Ljubljansko Barje aquifer, and (IV) Brest and Iški vršaj aquifer.

**Figure 3:**
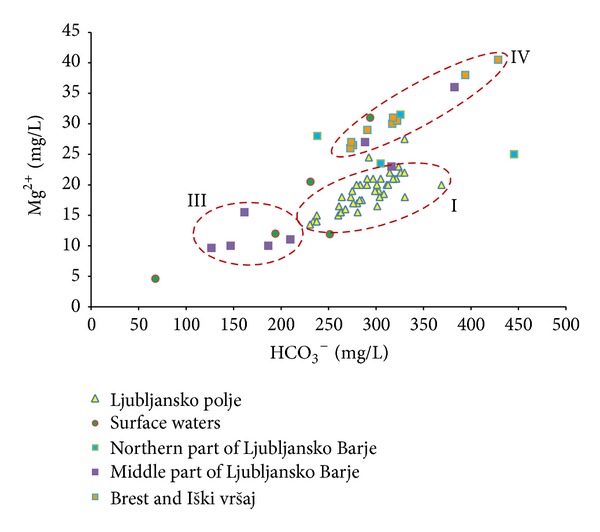
Relationship between HCO_3_^−^ and Mg^2+^ in groundwater of Ljubljansko polje and Ljubljansko Barje aquifers. Roman numerals represent groups of groundwater from (I) Ljubljansko polje aquifer, (III) middle part of Ljubljansko Barje aquifer, and (IV) Brest and Iški vršaj aquifer.

As a first step, groundwaters of both aquifers were separated into 4 main groups according to their hydrogeological and geographical position of sampling points (Figures 1–3):

- groundwater of Ljubljansko polje,
- groundwater of northern part of Ljubljansko Barje,
- groundwater of middle part of Ljubljansko Barje,
- groundwater of southern part of Ljubljansko Barje—Brest and Iški vršaj.

Table 1 shows estimates of several chemical parameters in groundwater of Ljubljansko polje and Ljubljansko Barje aquifers.

Figures 2 and 3 show the relationship between HCO_3_^−^/Ca^2+^ and HCO_3_^−^/Mg^2+^ in the groundwater. It is shown that groundwater from Ljubljansko polje aquifer (I) and Brest and Iški vršaj aquifers (IV) has the highest mineralization. At similar concentration of HCO_3_^−^, the groundwater of Ljubljansko polje aquifer has higher concentration of calcium and lower concentration of magnesium than that of Brest and Iški vršaj aquifer. The difference is approximately 20 to 30 mg/L for both chemical parameters.

We can interpret these results in the way that groundwater of Brest and Iški vršaj aquifer reflects the lithological characteristics of the Krimsko-Mokriško hills (1100 m  a.s.l.), which represents the main recharge area of the aquifer. The increased concentration of magnesium in the groundwater results from a significant amount of dolomites in the area. Similar hydrochemical characteristics are also indicated for groundwater of northern part of Ljubljansko Barje aquifer (II), but results are more scattered and do not form a clearly expressed group; therefore those sampling points are not enclosed in a dashed ellipse marked as (II). This groundwater discharges to Ljubljanica River and to Ljubljansko polje aquifer through the narrow between Grajski hrib, Rožnik, and Dravlje valley.

The groundwater of middle part of Ljubljansko Barje aquifer (III) represents infiltrated water from Gradaščica River recharge area, and it lies below impermeable clay layers on the surface. Main lithologies are Carboniferous and Permian shale and sandstone which are poor in carbonates, while the Mesozoic limestone and dolomites represent only a minor part. This is the reason for significantly lower groundwater mineralization. Other sampling points lie near sampling points at Brest and Iški vršaj aquifer and have similar groundwater carbonate characteristics, suggesting that recharge from carbonate southern periphery of the aquifer dominate.

**Figure 4:**
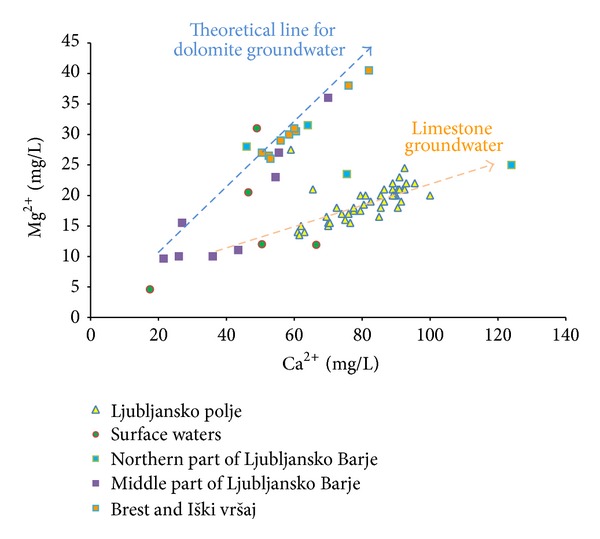
Relationship between Ca^2+^ and Mg^2+^ in groundwater of Ljubljansko polje and Ljubljansko Barje aquifers.

Figure 4 shows relationship between Ca^2+^ and Mg^2+^ in groundwater of both aquifers. Groundwaters are separated into 2 groups based on their recharge areas. The graph shows moderate correlation between Ca^2+^ and Mg^2+^ (*r* ^2^ = 0.30; *y* = 0.1454*x* + 7.104) for groundwater with dominating limestone in recharge areas. This group comprises groundwater from Ljubljansko polje aquifer and surface waters of Ljubljanica and Sava Rivers. Groundwater from Brest and Iški vršaj aquifer is classified as typical dolomite groundwater, as those sampling points lie near theoretical line for dolomite groundwater, calculated based on the molar ratio Ca/Mg equal to 1 [10].

**Figure 5:**
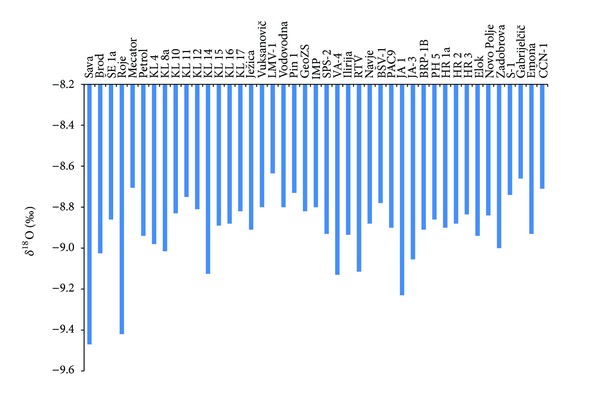
Isotopic composition of oxygen in groundwater of Ljubljansko polje aquifer.

Groundwater of the aquifers can also be separated based on *δ* ^18^O in groundwater.  Figure 5 shows basic characteristics of *δ* ^18^O in groundwater of Ljubljansko polje aquifer. Most of *δ* ^18^O values vary between −8.6 and −9.2‰. Sava River is depleted with *δ* ^18^O due to its mainly mountainous recharge area. Pronounced depletion with *δ* ^18^O was also detected at sampling locations Roje and JA 1, which indicates the predominance of drainage of Sava River to the aquifer.

**Figure 6:**
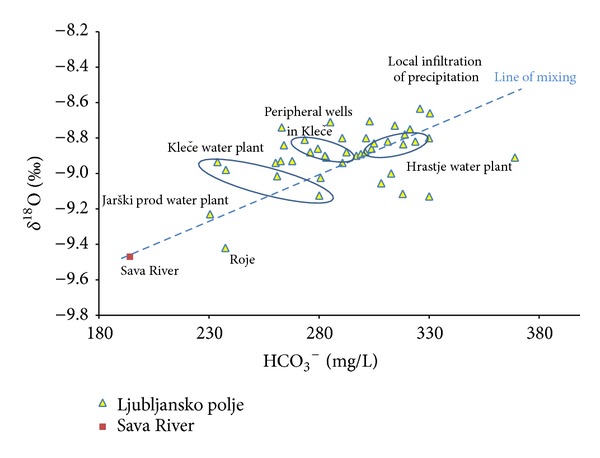
Relationship between HCO_3_^−^ and isotopic composition of oxygen (*δ* ^18^O) in groundwater of Ljubljansko polje and line of mixing of groundwaters of different origin.

Figure 6 shows two sources recharging the groundwater of Ljubljansko polje aquifer: infiltration of local precipitations and drainage of Sava River water with recharge area mainly in Julian Alps and Karavanke mountains. Mean value of oxygen isotopic composition (*δ* ^18^O) of Sava River water is −9.5‰, and −8.51‰  in monthly precipitation at observation point at Geological Survey in Ljubljana. The groundwater recharged from Sava River water has lower HCO_3_^−^ and has less dissolved carbonate compared to waters recharged from local precipitation. Jarški prod water plant and Roje were found to contain mainly higher proportion of Sava River water. These sampling points lie along the Sava River, where the influence of recharged water from Sava River is important. Moreover, groundwater flow from Sava River also dominates in the central part of the water plant Kleče, while in peripheral wells of Kleče and Hrastje water plants the influence of local precipitations prevails.

**Figure 7:**
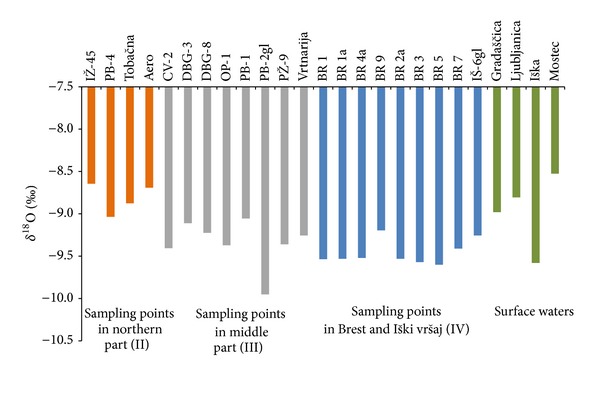
Isotopic composition of oxygen in groundwater and surface waters of Ljubljansko Barje aquifer.

Figure 7 shows isotopic characteristics of groundwater of Ljubljansko Barje aquifer. Most of the *δ* ^18^O values vary between −9.0 and −9.5‰. Groundwater from northern part of the aquifer is more enriched in oxygen-18 than that from other aquifers. This is due to considerable influence of local precipitations on this open aquifer. Values for groundwater *δ* ^18^O in this part vary between −8.6 and −9.0‰.

Based on *δ* ^18^O, water from Ljubljanica and Gradaščica Rivers stand out. Their recharge area extends far beyond our research area and its groundwater is enriched in oxygen-18 due to lower altitude of recharge area. Similar observations were noticed in Mostec Stream, its water *δ* ^18^O varying between −8.5 and −9.0‰.

Since main recharge area of Ljubljansko Barje aquifer is the Krimsko-Mokriško hills with mean altitude of 1100  a.s.l., groundwater is depleted in oxygen-18 due to the altitude effect.

Groundwater from Iški vršaj aquifer, sampled in wells located northeast of Iška River (BR-7 and BR-9), is more enriched in oxygen-18. This is due to influence of local precipitations which are more enriched in oxygen-18 compared to precipitations which fall at Krimsko-Mokriško hills. As mentioned earlier, the chemical composition of local precipitation is reflected in groundwater chemical composition in Brest and Iški vršaj aquifer. Groundwater *δ* ^18^O varies between −9.2 and −9.6‰.

**Figure 8:**
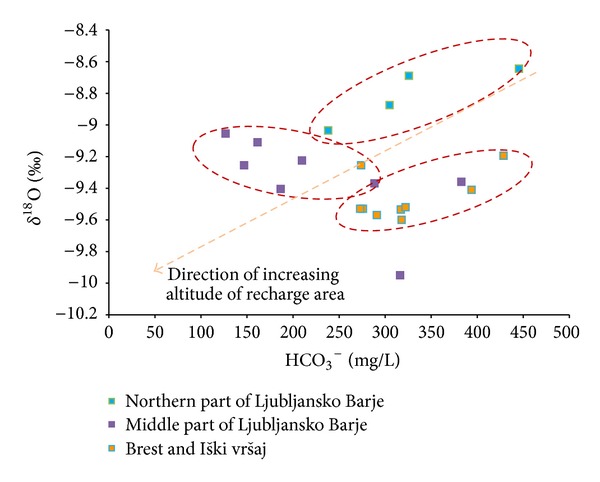
Relationship between HCO_3_^−^ and isotopic composition of oxygen (*δ* ^18^O) in groundwater of Ljubljansko polje and Ljubljansko Barje aquifers. Arrow shows direction of increasing altitude of recharge area.

Figure 8 clearly demonstrates three main groups of groundwater: that from Brest and Iški vršaj aquifer, from middle part of Ljubljansko Barje aquifer, and from northern part of Ljubljansko Barje aquifer.

Groundwater from Brest and Iški vršaj aquifer and groundwater from northern part of Ljubljansko Barje aquifer show similar positive correlation between *δ* ^18^O and HCO_3_^−^. Similar dependence is observed in many groundwaters in Slovenia. The reason lies in the dependence of both parameters on the altitude of recharge areas of aquifers. The *δ* ^18^O is influenced by altitude isotopic effect in precipitation [11], which is, for the continental part of Slovenia, −0.28‰  *δ* ^18^O/100 m [28], while the content of HCO_3_^−^ in groundwater is influenced by lower partial pressure of CO_2_ in the soil due to colder and shallower soils at higher altitudes [29].

**Figure 9:**
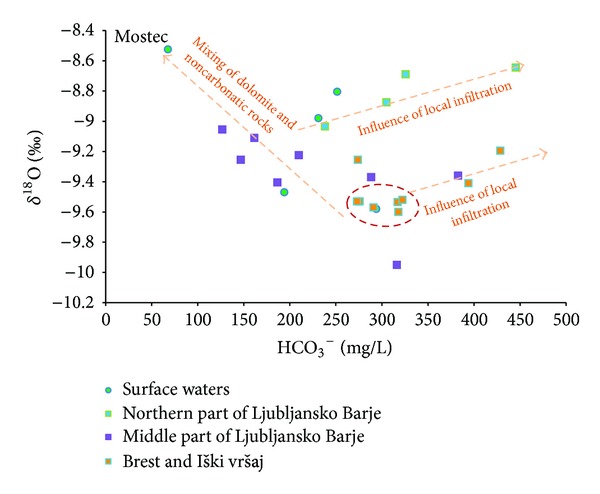
Interpretation of relationship between HCO_3_^−^ and isotopic composition of oxygen (*δ* ^18^O) in groundwater of Ljubljansko polje and Ljubljansko Barje aquifers.

For these reasons, the measured values shown in the graph above (Figure 8) can be interpreted as follows: more negative values of *δ* ^18^O and lower concentration of HCO_3_^−^ represent groundwater with higher recharge areas, while more positive values of *δ* ^18^O and higher concentration of HCO_3_^−^ represent groundwater with lower recharge areas of aquifer. The values in graph are plotted as an elongated cloud due to mixing of groundwater of different origin. The graph shows that groundwater from Ljubljansko Barje aquifer is recharged from higher altitudes from Krimsko-Mokriško hills and Polhograjsko hills (on the graph 8, below left), while values right above mostly show local lowland infiltration of precipitation on aquifer itself. Arrow on graph above shows known general direction of increasing altitude of recharge area based on data observed. Dependences described above are also shown in Figure 9. In this context, the question arises, why does the groundwater from middle part of Ljubljansko Barje aquifer deviate in chemical and isotopic composition compared to groundwater from northern part of Ljubljansko Barje aquifer and Iški vršaj aquifer?

Different hydrochemical characteristics of groundwater of middle part of Ljubljansko Barje aquifer can be interpreted by mixing of groundwater from two different origins: recharge from carbonate Krimsko-Mokriško hills in the southern periphery of the aquifer (*r* ^2^ = 0.27; *y* = 0.0014*x* − 9.8956) and recharge from noncarbonatic northwestern periphery of aquifer, where Carboniferous and Permian quartz sandstones and shales dominate (*r* ^2^ = 0.77; *y* = 0.0018*x* − 9.4103). Recharge area from Mostec stream mostly consists of those lithologies. In favor of such interpretation is the probable line of mixing of the plotted groundwater results from middle part of Ljubljansko Barje aquifer, which is oriented towards measured values of water from the Mostec stream.

Age and residence time of groundwater in aquifer can be estimated on the basis of tritium activity in groundwater, modeled by groundwater tritium exponential model [14]. The inputs for model are the data on tritium activity in precipitation at observation point at Geological Survey in Ljubljana which is now around 6 tritium units (TU). The measured tritium activities in precipitation are comparable to the tritium activities from the past researches in Slovenia and neighboring countries. Vreča et al. [28] present the mean annual tritium activity in Ljubljana and Zagreb precipitation in the period 1996–2004 as about 9 TU. Long-term tritium records for the continental stations Ljubljana and Zagreb showed that in the past mean annual tritium activity in precipitation decreased continuously after reaching a global atmospheric maximum in 1963 due to thermonuclear bomb-tests [30, 31].

**Figure 10:**
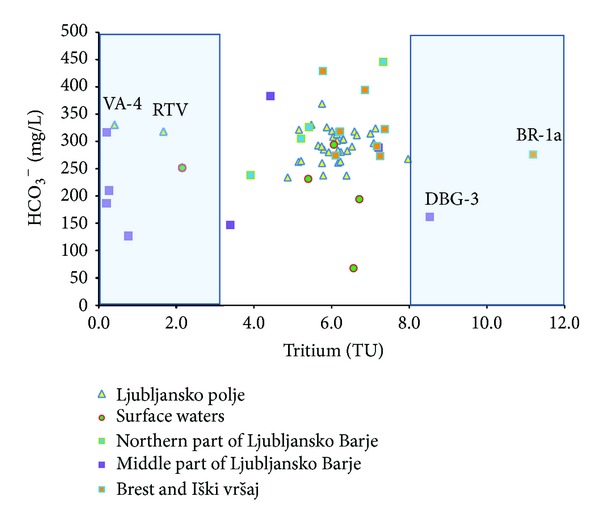
Relationship between HCO_3_^−^ and tritium in groundwater of Ljubljansko polje and Ljubljansko Barje aquifers.

Figure 10 shows results of measurements of tritium activity in groundwater of the aquifers. Most of the tritium activity in groundwater of Ljubljansko polje aquifer range between 5 and 7 TU and in groundwater of Ljubljansko Barje aquifer between 4 and 8 TU, and can therefore both be classified as “modern waters” [28, 32].

Groundwater at sampling locations VA-4 and RTV in Ljubljansko polje aquifer and groundwater from middle part of Ljubljansko Barje aquifer have tritium activity below 2 TU, which indicates “submodern groundwater” with residence time more than 50 years [32, 33]. Decrease in tritium activity in those waters is a result of radioactive decay in a closed aquifer structure, where the flow of groundwater is very slow. These could be due to lack of communication of the aquifer with surface; otherwise the tritium activity would be higher.

At sampling location BR 1a in Brest and Iški vršaj aquifer the tritium activity is significantly increased to 11 TU. Similarly elevated tritium activity of 9 TU has also been found in middle part of Ljubljansko Barje aquifer at sampling location DBG-3. Such values are higher than in current average precipitation, therefore according to tritium isotopic age model of groundwater these water can be classified as “older water”, with residence time between 10 and 50 years. In this water, increased tritium activity indicates “bomb tritium” from nuclear experiments in 1960s [14]. Pezdič [15] presented similar results where measured tritium activities in groundwater, springs, and surface rivers of Ljubljansko Barje reach 13.4 TU, with long-term averages (*n* = 13 years) 17.5 TU. Also Kožar Logar et al. [34] presented tritium activities in groundwater of Ljubljanko Barje with maximum 16 TU.

### 3.2. Interaction of Groundwater between Ljubljansko Barje and Ljubljansko Polje Aquifers

Interaction of groundwater between aquifers can be estimated based on carbonate characteristics (Ca/Mg molar ratio and HCO_3_^−^) of groundwater, as groundwater from Ljubljansko Barje aquifer also partly recharges Ljubljansko polje aquifer.

**Figure 11:**
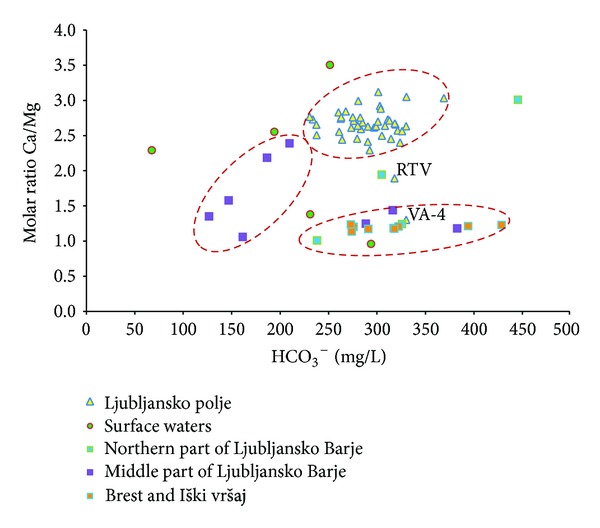
Relationship between HCO_3_^−^ and Ca/Mg molar ratio in groundwater of Ljubljansko polje and Ljubljansko Barje aquifers.

Figure 11 shows the relationship between Ca/Mg molar ratio and HCO_3_^−^. It is clearly demonstrated that recharge areas of Ljubljansko Barje aquifer are mainly composed of dolomite as molar ratio Ca/Mg ranges between 1.0 and 1.5 [10]. Higher values can be found in groundwater of Brest and Iški vršaj aquifer and also in northern part of Ljubljansko Barje aquifer, where local infiltration of precipitation to groundwater influence the composition of groundwater.

Values of Ca/Mg molar ratio in groundwater of Ljubljansko polje aquifer range between 2.5 and 3.0 which reflect the limestone predomination over dolomite in recharge area of the aquifer [10]. Sampling locations VA-4 and RTV situated near boundary between those two aquifers are exceptions with values of molar ratio between 1.3 and 1.9. Groundwater of Ljubljansko Barje aquifer has a higher proportion of magnesium; therefore we can assume that groundwater from Ljubljansko Barje aquifer occurs at both sampling locations. However, at similar concentrations of HCO_3_^−^ in groundwater, the value of molar ratio Ca/Mg at RTV is higher than at VA-4. This can be interpreted with the location of RTV in Ljubljansko polje aquifer, which lies further away from the border of the two aquifers where the influence on groundwater of Ljubljansko Barje is lower. We can estimate that groundwater in sampling point RTV contains approximately 50% of groundwater from Ljubljansko Barje, while at VA-4 this percentage is almost 100%.

## 4. Conclusions

The objective of the presented study was to obtain new data on mixing and dynamics of groundwater in Ljubljansko polje and Ljubljansko Barje aquifers based on hydrochemical and isotopical data of groundwater. Obtained data were used to identify differences in recharging areas of those two aquifers. Groundwaters were separated into 4 groups.

Groundwater of Ljubljansko polje aquifer with higher calcium content is recharged from infiltration of local precipitations and drainage of Sava River water having recharge area mainly in Julian Alps and Karavanke mountains where limestone rocks dominate. Sava River water is depleted with *δ* ^18^O due to its recharge area mainly from higher altitude areas. Pronounced depletion of *δ* ^18^O was also detected at sampling locations in which the influence of recharged water from Sava River is important, while more positive values of *δ* ^18^O were recorded at sampling locations where local infiltration of precipitation dominates, further away from the Sava River.

Groundwater of northern part of Ljubljansko Barje aquifer discharges to Ljubljanica River and to Ljubljansko polje aquifer through the narrow between Grajski hrib, Rožnik, and Dravlje valley. Groundwater has similar hydrochemical characteristics as groundwater of Brest and Iški vršaj aquifer where the increased concentration of magnesium results from significant proportion of dolomites in the recharge area. The groundwater is more enriched in *δ* ^18^O than in other part of the aquifer due to infiltration of local precipitations in lowland that have great influence on this open aquifer.

Groundwater of middle part of Ljubljansko Barje lies below surface cover of impermeable clay and has significantly lower mineralization due to less permeable low-carbonate Carboniferous and Permian rocks in their recharge area. In this part of the aquifer occurs the mixing of groundwaters with carbonate dolomite waters from southern periphery of aquifer on one side and noncarbonate groundwater from western periphery of Ljubljansko Barje aquifer recharged from Gradaščica River on the other side.

Groundwater from Brest and Iški vršaj aquifer is classified as typical dolomite groundwater due to increased magnesium content. Groundwater reflects the lithological characteristics of the Krimsko-Mokriško hills, which represents the main recharge area of the aquifer. Also the depletion in *δ* ^18^O in groundwater was indicated as a result of the isotope altitude effect.

Additionally, also the groundwater age and residence time were estimated according to the tritium activity measured in precipitation and groundwater. Long-term tritium records showed that in the past mean annual tritium activity in precipitation decreased continuously after reaching a global atmospheric maximum in 1963 due to thermonuclear bomb-tests. The measured tritium activities in precipitation are comparable to the tritium activities from the past researches in Slovenia and neighboring countries. Reported mean annual tritium activity in precipitation in the period 1996–2004 was about 9 TU. Tritium activity in precipitation at observation point at Geological Survey in Ljubljana is now around 6 tritium units (TU).

Most groundwaters of the two aquifers are classified as “modern waters” with residence time of up to 10 years. Groundwater sampled on the border between the two aquifers and groundwater from middle part of Ljubljansko Barje aquifer have tritium activity below 2 TU, indicating “submodern groundwater” with residence time of more than 50 years. Decrease in tritium activity in those waters is a result of radioactive decay in a closed aquifer structure. At sampling location BR 1a in Brest and Iški vršaj aquifer the measured tritium activity was up to 11 TU. A similarly increased tritium activity with the value of 9 TU has also been found in middle part of Ljubljansko Barje aquifer at sampling location DBG-3. These waters can be classified as “older water” with residence time between 10 and 50 years. In these waters the increased tritium activity indicates “bomb tritium” from nuclear experiments in 1960s.

Interaction of groundwater between Ljubljansko polje and Ljubljansko Barje aquifers was estimated also based on carbonate characteristics of groundwater. Values of Ca/Mg molar ratio range between 2.5 and 3.0 in groundwater of Ljubljansko polje aquifer which indicates predominance of limestone in recharge area. On the other hand, the values of Ca/Mg molar ratio in groundwater of Ljubljansko Barje aquifer are lower and range between 1.0 and 1.5, indicating dolomite as a dominant rock in the recharge area. Values of molar ratio in groundwater at sampling points VA-4 and RTV are 1.3 and 1.9. They lie near boundary of those two aquifers where influence of groundwater of Ljubljansko Barje is important since it drains in aquifer of Ljubljansko polje. We estimated the extent of this influence on both sampling points. In VA-4, which lies directly on the border between the two aquifers, the influence is almost 100%. In sampling point RTV the influence is approximately 50%, as it lies away from the border, in the inner part of the Ljubljansko polje aquifer.

Quantitative results obtained represent the basis for improvement of hydrogeological conceptual models of both aquifers, which will enable more accurate simulation of the groundwater dynamics and transport of pollutants in the aquifer. Obtained data will also provide the basis for further planning of exploitation of groundwater from both aquifers for drinking water supply and for planning measures for protection of water resources.

## Figures and Tables

**Table 1:** Descriptive statistics of relevant chemical and isotopic parameters in groundwater of Ljubljansko polje and Ljubljansko Barje aquifers.

			Molar ratio			
	Ca^2+^	Mg^2+^	Ca/Mg	HCO_3_^−^	*δ* ^18^O	Tritium
	mg/L	mg/L	/	mg/L	‰	TU
Ljubljansko polje aquifer (*n* = 43)						
Arithmetic mean	80.9	18.9	2.60	292	−8.9	5.84
Standard deviation	10.7	2.9	0.3	29	0.2	1.3
Median	82.5	19	2.7	292	−8.9	6.1
Minimum	59.0	13.5	1.30	230	−9.4	0.41
Maximum	100.0	27.5	3.12	369	−8.6	7.97
Northern part of Ljubljansko Barje aquifer (*n* = 4)						
Arithmetic mean	77.4	27.0	1.8	329	−8.8	5.5
Standard deviation	28.9	3.1	0.8	75	0.2	1.2
Median	69.8	26.5	1.6	315	−8.8	5.3
Minimum	46.0	23.5	1.0	238	−9.0	3.9
Maximum	124.0	31.5	3.0	446	−8.6	7.3
Middle part of Ljubljansko Barje aquifer (*n* = 8)						
Arithmetic mean	41.8	17.8	1.6	227	−9.3	3.1
Standard deviation	16.1	9.2	0.5	86	0.3	3.1
Median	39.8	13.3	1.4	198	−9.3	2.1
Minimum	21.5	9.7	1.1	127	−10.0	0.2
Maximum	70.0	36.0	2.4	383	−9.1	8.5
Brest and Iški vršaj in southern part of Ljubljansko Barje aquifer (*n* = 9)						
Arithmetic mean	61.0	30.9	1.2	322	−9.5	7.1
Standard deviation	10.2	4.8	0.0	52	0.1	1.5
Median	58.5	30.0	1.2	317	−9.5	6.9
Minimum	50.5	26.0	1.1	273	−9.6	5.8
Maximum	82.0	40.5	1.2	429	−9.2	11.2
Surface waters (*n* = 5)						
Arithmetic mean	46.0	16.0	2.1	208	−9.1	5.4
Standard deviation	15.9	9.0	0.9	77	0.4	1.7
Median	49.0	12.0	2.3	231	−9.0	6.1
Minimum	17.5	4.6	1.0	68	−9.6	2.2
Maximum	66.5	31.0	3.5	294	−8.5	6.6
